# The Moral Foundations of Desired Cultural Tightness

**DOI:** 10.3389/fpsyg.2022.739579

**Published:** 2022-04-19

**Authors:** Daniela Di Santo, Michele J. Gelfand, Conrad Baldner, Antonio Pierro

**Affiliations:** ^1^Department of Social and Developmental Psychology, Sapienza University of Rome, Rome, Italy; ^2^Stanford Graduate School of Business, Stanford, CA, United States

**Keywords:** cultural tightness, moral foundations, morality, ecological threat, desired tightness

## Abstract

People vary on their desire for strict norms, and the moral underpinnings of these differences have yet to be explored. The current research examined whether and how moral beliefs held by individuals would affect the extent to which they want their country to be tight (i.e., having strict social norms) or loose (i.e., having more permissive social norms). In particular, the effects of the “binding” and “individualizing” foundations, which are moral beliefs focused on the importance of groups and individuals, respectively, were examined. We hypothesized that the binding foundations could predict people’s desire for cultural tightness. We also hypothesized that the perception that one’s society is threatened may drive this effect. Three studies were conducted using both cross-sectional (Studies 1 and 3) and two-wave (Study 2) designs. Demographic variables and participants’ political orientation effects were controlled. In Study 1, only the binding foundations significantly predicted higher desired tightness. In Study 2, binding foundations predicted desired tightness measured at follow-up. In Study 3, the positive effect of perceived threat on desired tightness *via* the binding foundations was confirmed. From additional within-paper analyses we also have some evidence of significant relationships, albeit unstable across studies, between desired tightness and individualizing foundations.

## Introduction

### “Tightness” and “Looseness” at the Cultural and Individual Level

Let us imagine two people debating the social norms of their country, the first calling for stricter rules and punishments for transgressors and the second for more flexibility and tolerance. The former calls for more “tightness,” the latter for more “looseness.” Tightness and looseness reflect the strength of social norms in ones’ environment and the degree of sanctioning within societies (Gelfand et al., 2006; Gelfand, 2018). Tight cultures have stronger social norms and greater sanctioning of deviant behavior (Gelfand et al., 2011). Conversely, loose cultures have weaker social norms and little sanctioning of deviant behavior (Gelfand et al., 2011). Tightness–looseness is part of a multilevel system that comprises ecological, historical, and institutional factors, along with everyday situations and psychological processes (Gelfand et al., 2011; Gelfand, 2018). Differences in tightness–looseness often arise from differences in ecological and historical conditions, where tight countries historically experienced more ecological threats (i.e., resource scarcity, higher disease prevalence, territorial invasions and conflicts, high population density, environmental threats) compared to loose countries (Gelfand et al., 2011; Harrington and Gelfand, 2014). For cultures that face many threats, tight social norms are adaptive as they promote the coordination that is necessary for survival (Harrington and Gelfand, 2014; Gelfand, 2018). Tightness–looseness is manifested in both societal institutions (e.g., autocracy) and everyday recurring situations (e.g., situational strength). Tight cultures, for example, tend to feature strong (vs. weak) situations, characterized by more situational constraint, potential censorship, a narrower range of appropriate behaviors, and less individual discretion (Gelfand et al., 2011). Individuals who are chronically exposed to these situations experience greater behavioral constraint and risk of punishments for norm violations. Therefore, individuals’ psychological processes often adapt to meet cultures’ demands (Gelfand et al., 2011). For example, living in a tight country is associated with having greater caution, regulatory strength, and dutifulness, among others (see Gelfand et al., 2011).

While people are all shaped by their cultural context, at the individual level, the degree to which individuals desire that their country be tight can vary from person to person regardless of country-level tightness. As argued in Jackson et al. (2019), desire for cultural tightness is not the same as living in a tight society, because a person may endorse a culture that is different than the one they are living in. For example, based on the results of Gelfand et al. (2011) it would seem that Italy is not a particularly tight culture, having, as a whole, a national tightness score (6.8) nearly equivalent to the average (6.5). However, even for participants who are from loose cultures, the relevance of a threat has been shown to increase the desire for tight social norms (Caluori et al., 2017; Jackson et al., 2019; see also Gelfand et al., 2017). In this article, we aimed to look at some factors that can potentially trigger desired tightness.

We specifically looked at the moral beliefs held by individuals that could drive their desire for tight culture. More in particular, the effects of the “binding” and “individualizing” foundations, which are moral beliefs focused on the importance of groups and individuals, respectively, were examined. We expected that the concern for the group as reflected by binding foundations would stimulate people’s desire for cultural tightness; if individuals perceive that their “group” (e.g., their larger culture) is threatened then they may approve of tight rules, and consequent punishments for breaking them, in order to protect that “group.” On the other hand, there is a less strong case for the role of the individualizing foundations: individuals high in these foundations may dislike tight, impersonal rules, but they might also favor these rules if they can protect others. We also examined whether these moral beliefs, which can drive desires for tight norms, could be motivated by a perceived threat. In other words, we expect that the binding foundations would mediate the relationship between threat and desire for tightness. We assume that the threat may drive the endorsement of binding foundations, rather than the other way around, because the literature has found that uncertainties of various kinds, such as perceptions of social dangers (Van Leeuwen and Park, 2009) and COVID-19 concern (Bianco et al., 2021), stimulate the endorsement of the binding moral foundations; accordingly, we hypothesize that it is the perceived socio-ecological threat (as a source of concern and uncertainty) that can stimulate the endorsement of binding moral foundations, and, subsequently, the desired tightness. In the following, before specifying our hypotheses, we briefly summarize the moral beliefs described in the moral foundations theory (Graham et al., 2011), and then, we explain the rationale for the hypotheses.

### Moral Foundation Theory

According to Haidt (2012), moral intuitions underlie the moral systems developed by cultures (see also Haidt and Joseph, 2004). Moral foundations theory (Haidt and Graham, 2007; Graham et al., 2009, 2011; Haidt, 2012) asserts that people make moral judgments of right and wrong through moral intuitions, and identifies two broad and superordinate categories of moral foundations: the “individualizing” foundations of care and fairness, and the “binding” foundations of authority, loyalty to the in-group, and purity. Accordingly, in the present paper, we focused primarily, also for reasons of parsimony, on the two superordinate categories of moral foundations conceptualized by Graham et al. (2011), and, more specifically, on the binding foundations.

While the individualizing foundations are primarily concerned with protecting the rights and freedoms of individual people, the binding foundations are primarily concerned with preserving larger groups (e.g., organizations but also the overall culture) through duties, loyalty, and (social and physical) purity. Extensive research has examined the link between moral concerns and political ideology (e.g., Haidt and Graham, 2007; Graham et al., 2009; Federico et al., 2013; Weber and Federico, 2013). Findings have shown that political conservatives and liberals weigh moral concerns differently. Conservatives tend to weigh binding and individualizing foundations equally, while liberals prioritize the individualizing foundations (e.g., Graham et al., 2009). Research has also found that emphasis on the binding moral foundations relative to the individualizing foundations underlies conservative ideology, which is characterized by resistance to change and tolerance of inequality (Haidt et al., 2009; Van Leeuwen and Park, 2009). The binding foundations uphold conservative values by preserving the integrity of social groups and structures through obedience to hierarchy and conformity to traditions (authority), fidelity and duties toward one’s group (loyalty), and respect for God, natural laws, and constraints for baser instincts (purity; Haidt and Graham, 2007). Consistently, the endorsement of binding foundations has been shown to prompt more conservative attitudes on various ideological and social issues, such as abortion, same-sex marriage, immigration, and crime, among others (e.g., Koleva et al., 2012; Silver and Silver, 2017; Baldner et al., 2020).

### The Present Research

Binding moral foundations may affect individuals’ belief about the importance of upholding of tight social norms. We examine this question in the current research, that is, whether moral priorities guide people in upholding patterns of social norms. To our knowledge, no research has assessed the impact of morality on the people’s desire for cultural tightness. We hypothesize that the binding foundations predict individuals’ desire for tightness; the binding foundations value social institutions over individuals (Haidt and Graham, 2007) and emphasize community bonding and duty toward one’s group, obedience and respect for authority, social hierarchies, traditions (i.e., social norms), and self-regulation of basic instincts (constraints). For example, endorsement of binding values, compared with individualizing values, elicited higher levels of punitiveness for crimes, through the perception that the crime was perpetrated against society (Silver and Silver, 2017). Similarly, binding foundations have been found to mediate the relationship between closed mindedness and punishment with the intent to deter future crime (Giacomantonio et al., 2017). In general, the binding foundations reflect a preference for group cohesion, which could be attained through a system of strong social norms and punishments. In other words, it is possible that moral concern for the welfare of one’s ingroup may motivate the desire for a tight culture. We thus predict that binding foundations will lead people to increasingly favor cultural tightness. We initially tested this hypothesis in two studies. Furthermore, a tight cultural system can be perceived to facilitate societal survival, through the perceptions of increased maintenance of social order (at the cost of severe punishment; Gelfand, 2018) and of better coordination in the face of threats (Gelfand et al., 2011; Harrington and Gelfand, 2014). Therefore, a perceived threat to one’s group should raise moral concern for the group and the desire to defend it through stricter regulations. We tested this idea in a third study using a mediation model where perceived ecological threat can increase the binding moral concerns, which can, in turn, increase desire for a tight culture.

**Figure 1:**
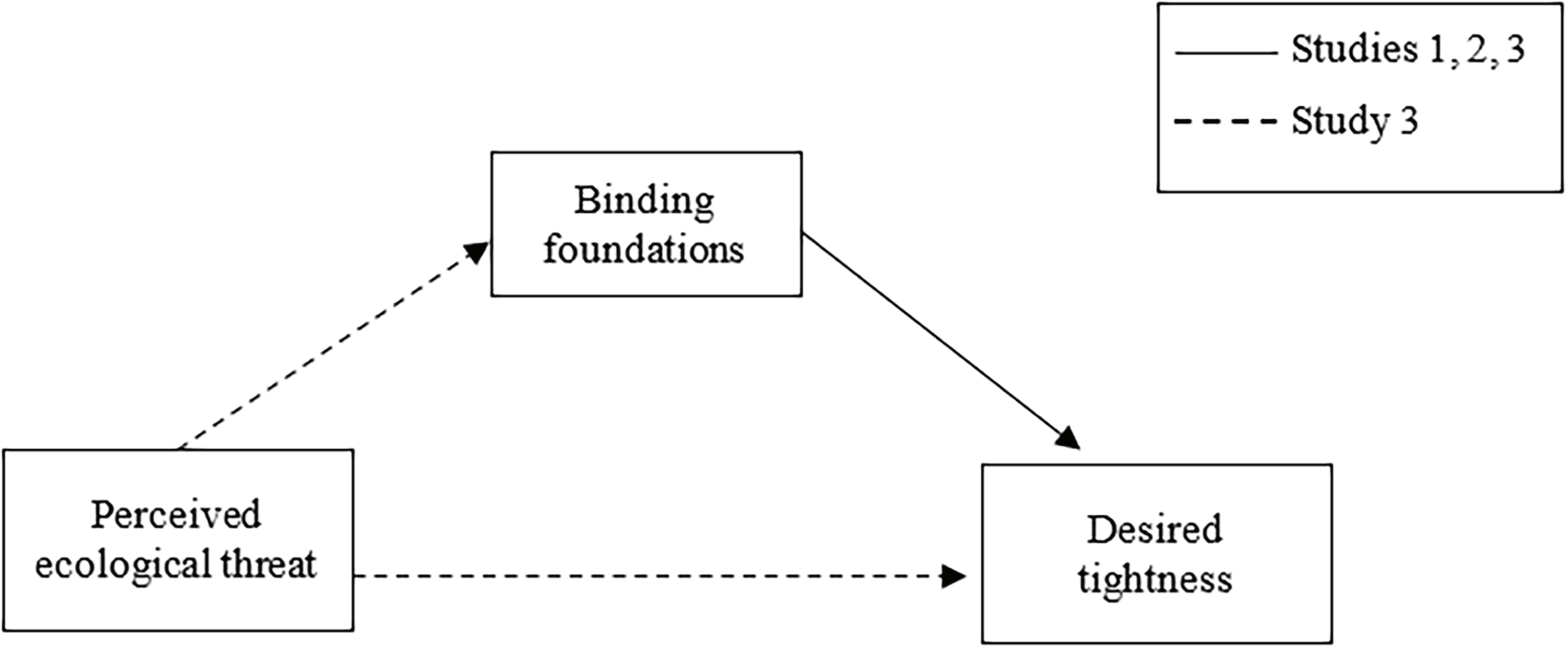
Hypothetical model of the studies.

In summary, three studies were conducted in Italy, using both cross-sectional (studies 1 and 3) and two-wave (study 2) designs, described below. We expected that the binding foundations would predict desired tightness; given the state of the literature on moral foundations, we did not make any hypotheses for the individualizing foundations. However, the effect of the individualizing foundations on desired tightness will also be explored and will be reported for readers. Our hypotheses were specifically the following and were summarized in Figure 1:

- Binding foundations will lead people to increasingly favor cultural tightness.
- Binding foundations measured at one time will lead people to increasingly favor subsequent cultural tightness.
- Perceived threat will lead people to increasingly favor cultural tightness through the endorsement of the binding foundations.

## Study 1

### Method

#### Participants and Procedure

A total of 332 university students (79% females; *M_age_* = 22.92 years, *SD_age_* = 4.159) voluntarily agreed to participate in the study, gave their informed consent, and completed an online survey with the following measures of moral foundations and desired cultural tightness. Participants also indicated their gender, age, and political orientation (on a 7-point scale ranging from 1 = *“left-wing”* to 7 = “*right-wing*”). All study materials were presented in Italian. According to a sensitivity analysis, and given a sample size of 332 and *α* and power set to 0.05 and 0.80, respectively, and with five predictors, we had the power to detect an effect of *f^2^* = 0.039.

##### Moral Foundations

We administered to participants the Italian version of the Moral Foundations Questionnaire (MFQ; Graham et al., 2013b; see Bobbio et al., 2011, for an analysis of the psychometric properties of the Italian version), a 30-item measure of the extent to which people hold the binding moral foundations (with subfactors of Ingroup/loyalty, Authority/respect, and Purity/sanctity) and the individualizing foundations (with subfactors of Harm/care and Fairness/reciprocity). The questionnaire consisted of two parts: in the first part, participants rated how relevant (from “1 = Not at all relevant” to “6 = Extremely relevant”) each of 15 sources of information were to them when making moral judgments (e.g., “Whether or not someone showed a lack of respect for authority,” “Whether or not someone cared for someone weak or vulnerable”); in the second part, participants rated their agreement (from “1 = Strongly disagree” to “6 = Strongly agree”) with 15 moral statements (e.g., “Compassion for those who are suffering is the most crucial virtue,” “If I were a soldier and disagreed with my commanding officer’s orders, I would obey anyway because that is my duty”).^1^ In line with the literature that used the broad categories of moral foundations (e.g., Wright and Baril, 2011; Harper and Rhodes, 2021), we collapsed the five moral foundations into the two higher-order variables, finding satisfactory internal reliability for both the binding (Cronbach’s *α* = 0.84) and individualizing (Cronbach’s *α* = 0.73) scales.

##### Desired Cultural Tightness Scale

We used the desire for cultural tightness scale developed by Jackson et al. (2019). Participants were asked to endorse an ending to nine incomplete statements concerning their desired country tightness on a 9-point scale (e.g., “My country is currently…” 1-Not Permissive Enough—9-Too Permissive or “Social norms in my country are…” 1-Too rigid—9-Too flexible; full scale is provided as Supplementary Material). The prevalence of high-anchored responses indicated higher desired tightness. The reliability of the scale was deemed satisfactory (Cronbach’s *α* = 0.80).

### Analyses and Results

A summary of the means, standard deviations, and correlations between variables is presented on Table 1. As can be seen, both the binding foundations [*r* = 0.274, *p* < 0.001, 95% CI (0.171, 0.370)] and the individualizing foundations [*r* = 0.128, *p* = 0.02, 95% CI (0.020, 0.232)] were positively correlated with desired tightness. However, the binding foundations were significantly more correlated with desired tightness (Hotelling’s test: *Z* = 2.291, *p* < 0.05) than were the individualizing foundations.

**Table 1:** Summary of means, standard deviations, and correlations between variables, Study 1.

	M(SD)	1	2	3	4	5	6
1. Gender	–	–					
2. Age	22.92(4.16)	0.031	–				
3. Political orientation	3.52(1.34)	−0.065	−0.239^***^	–			
4. Binding	3.81(0.69)	−0.008	−0.141^**^	0.286^***^	(0.84)		
5. Individualizing	4.93(0.56)	0.237^***^	0.074	−0.181^***^	0.293^***^	(0.73)	
6. Desired Tightness	6.13(1.02)	0.085	−0.064	0.273^***^	0.274^***^	0.128^*^	(0.80)

Binding, Binding foundations; Individualizing, Individualizing foundations. Gender was coded as 0 = Male, 1 = Female. ^**^p ≤ 0.01, and ^***^p ≤ 0.001; In parentheses (Cronbach’s alpha). N = 332.

Political orientation also correlated positively [*r* = 0.273, *p* < 0.001, 95% CI (0.170, 0.369)] with desired tightness, whereby right-wing people were more supportive of tightness. This result is consistent with other research that found a relationship between tightness and outcomes adjacent to political conservatism (see Harrington and Gelfand, 2014). Political orientation also correlated positively with the binding foundations [*r* = 0.286, *p* < 0.001, 95% CI (0.184, 0.381)] and negatively correlated [*r* = −0.181, *p* = 0.001, 95% CI (−0.283, −0.074)] with the individualizing foundations.

Next, we conducted a regression analysis with the moral foundations and control variables as predictors and the desired tightness as a dependent variable. As control variables we used demographics and political orientation; given the relationship between tightness and politically related outcomes (Harrington and Gelfand, 2014), we felt it was important to control the effect of political orientation on endorsement of more or less rigid norms and punishments.^2^ Results are presented in Table 2; coefficients are unstandardized. Political orientation predicted higher desired tightness (95% CI: 0.104, 0.275). Including control variables, the individualizing foundations did not predict desired tightness (95% CI: −0.022, 0.396). Importantly, the binding foundations predicted higher desired tightness (95% CI: 0.087, 0.423), even with our control variables. Our results showed that the binding foundations had a semipartial correlation of 0.154 with tightness; this is equivalent to an *f*^2^ of 0.027. Therefore, the effect of the binding foundations was not sufficiently large (i.e., *f*^2^ < 0.039) to be detected in Study 1. However, it could be possible to combine data from Study 1 with further studies, in order to assess if the effect of the binding foundations is sufficiently large given a larger sample (see the Supplementary Material).

**Table 2:** Predictive effects of the moral foundations and covariates on desired tightness, Study 1.

Predictors	*b*	*t*	*p*	95% CI
Gender	0.19	1.454	0.147	−0.07, 0.45
Age	0.002	0.184	0.854	−0.02, 0.03
Political orientation	0.19	4.365	<0.001	0.10, 0.27
Binding	0.25	2.991	0.003	0.09, 0.42
Individualizing	0.19	1.763	0.079	−0.02, 0.40
	*R*^2^ = 0.13, ^Adjusted^*R*^2^ = 0.12			

*Binding, Binding foundations; Individualizing, Individualizing foundations. Gender was coded as 0 = Male, 1 = Female. N = 332. Regression coefficients are unstandardized*.

### Discussion

We hypothesized that the binding foundations would predict the desire for one’s country to have strong social norms and greater sanctioning. The results of Study 1 provided preliminary support for this hypothesis. Our results also show that, although a positive correlation exists between desired tightness and the individualizing foundations, the individualizing foundations do not significantly predict desired tightness when other predictors are included. Due to the cross-sectional nature of this study, it was impossible to determine a causal relationship between the variables. Although linear regression cannot establish a causal relationship on an outcome variable, we used a two-wave design in Study 2 to observe whether moral foundations measured earlier would have predicted desired tightness measured later.

## Study 2

### Method

#### Participants and Procedure

A total of 117 university students completed the initial survey (Time 1). Of them, 111 responded to the second wave of the survey (response rate of 94.9%) and were considered as the final sample^3^ (65% females; *M_age_* = 24.27 years, *SD_age_* = 3.908). Participants voluntarily agreed to participate in the study, gave their informed consent, and completed a paper-and-pencil questionnaire with the same measures of binding (*α* = 0.86) and individualizing (*α* = 0.60) moral foundations^4^ and desired cultural tightness (*α* = 0.75) as in Study 1. They indicated their gender, age, and political orientation as in Study 1. Two months later, students received invitations to complete a follow-up questionnaire in exchange for course credits, which included the same measure of desired cultural tightness (*α* = 0.81) as Time 1.

### Analyses and Results

A summary of the means, standard deviations, and correlations between variables for Study 2 is presented on Table 3. Confirming results of Study 1, the binding foundations positively correlated (Table 3) with desired tightness at Time 1 [*r* = 0.504, *p* < 0.001, 95% CI (0.350, 0.631)] and at follow-up [Time 2; *r* = 0.537, *p* < 0.001, 95% CI (0.389, 0.657)]. The individualizing foundations did not significantly correlate with desired tightness at Time 1 [*r* = 0.171, *p* = 0.072, 95% CI (−0.016, 0.346)] or at follow-up [*r* = 0.100, *p* = 0.295, 95% CI (−0.088, 0.281)].

**Table 3:** Summary of means, standard deviations, and correlations between variables, Study 2.

	M(SD)	1	2	3	4	5	6	7
1. Gender	–	–						
2. Age	24.27(3.91)	−0.128	–					
3. Political orientation	3.11(1.07)	−0.014	−0.053	–				
4. Binding	3.22(0.69)	0.044	−0.047	0.437^***^	(0.86)			
5. Individualizing	4.74(0.45)	0.246^**^	0.151	−0.019	0.249^**^	(0.60)		
6. Desired Tightness at Time1	5.99(1.00)	0.123	−0.084	0.372^***^	0.504^***^	0.171	(0.75)	
7. Desired Tightness at Time 2	5.85(1.08)	0.232^*^	−0.160	0.441^***^	0.537^***^	0.100	0.710^***^	(0.81)

Binding, Binding foundations; Individualizing, Individualizing foundations. Gender was coded as 0 = Male, 1 = Female. ^*^p ≤ 0.05, ^**^p ≤ 0.01, and ^***^p ≤ 0.001; In parentheses (Cronbach’s alpha). N = 111.

Furthermore, confirming the results of Study 1, desired tightness correlated more strongly (Table 3) with the binding foundations than the individualizing foundations both at Time 1 (Hotelling’s test: *Z* = 3.098, *p* < 0.01) and at follow-up (Hotelling’s test: *Z* = 4.043, p < 0.01).

Finally, political orientation correlated positively with desired tightness at Time 1 [*r* = 0.372, *p* < 0.001, 95% CI (0.199, 0.522)] and at follow-up [*r* = 0.441, *p* < 0.001, 95% CI (0.277, 0.579)], with conservatives showing stronger desired tightness. Gender (codified as Male = 0, Female = 1) was not significantly related to desired tightness at Time 1 [*r* = 0.123, *p* = 0.200, 95% CI (−0.064, 0.302)], but at follow-up women were more likely to desire cultural tightness [*r* = 0.232, *p* = 0.014, 95% CI (0.047, 0.401)].

We then conducted a regression analysis with the moral foundations and our control variables (age, gender, and political orientation) measured at Time 1 as predictors and the desired tightness measured at follow-up as a dependent variable. We included desired tightness measured at Time 1 as a control variable to ensure that the effects of the predictors on desired tightness measured subsequently were not explained by their contemporaneous associations. Results are presented in Table 4; coefficients are unstandardized. The binding foundations positively predicted desired tightness at follow-up (95% CI: 0.088, 0.573). Gender and political orientation also positively predicted desired tightness at follow-up (95% CI: 0.088, 0.672 and 95% CI: 0.006, 0.290, respectively). As in Study 1, individualizing foundations did not significantly predict desired tightness at follow-up (95% CI: −0.496, 0.149).

**Table 4:** Predictive effects of the moral foundations and covariates on subsequent desired tightness, Study 2.

Predictors (Time 1)	*b*	*t*	*p*	95% CI
Gender	0.38	2.578	0.011	0.09, 0.67
Age	−0.02	−1.010	0.315	−0.05, 0.02
Political orientation	0.15	2.060	0.042	0.01, 0.29
Binding	0.33	2.703	0.008	0.09, 0.57
Individualizing	−0.17	−1.067	0.289	−0.50, 0.15
Desired Tightness	0.58	7.231	<0.001	0.42, 0.74
	*R*^2^ = 0.60, ^Adjusted^*R*^2^ = 0.58			

*Binding*, *Binding foundations; Individualizing, Individualizing foundations. Gender was coded as 0 = Male, 1 = Female. N = 111. Regression coefficients are unstandardized*.

### Discussion

In Study 2, we replicated the results of Study 1 using a two-wave design. We found again that the binding foundations measured at Time 1 positively predicted people’s desired tightness 2 months later, controlling for participants’ gender, age, political orientation, and individualizing foundations scores and desired tightness at Time 1. The individualizing foundations again did not significantly predict people’s desired tightness. At this point, we conducted a third study to test the idea (as shown earlier in Figure 1) that the endorsement of binding foundations and tightness could be motivated by a perceived threat to society; more specifically, we hypothesized that a strong moral concern for the preservation of one’s group could lead to a desire for stricter regulations in the face of a perceived socio-ecological threat. This mediation hypothesis was tested in Study 3, described below.

## Study 3

### Method

#### Participants and Procedure

Power analysis for detecting mediating effects using MedPower (Kenny, 2017) indicated that at least 252 participants were required to detect a small to medium indirect effect (i.e., partial *r* for all paths = 0.20) with sufficient power (0.80) at 0.05 alpha level. A total of 285 university students (58% females; *M_age_* = 22.55 years, *SD_age_* = 3.058) were recruited online (i.e., *via* Prolific Academic) and received monetary compensation for participating. Participants gave their informed consent and filled an online survey with the following measures. All study materials were presented in Italian.

##### Perceived Socio-Ecological Threat

Participants were asked to rate their concern about seven different ecological threats which are salient in Italy and that could undermine society (i.e., economic crisis, employment difficulties, international conflicts, terrorism, immigration-related phenomena, political instability, coronavirus), on a seven-point Likert scale ranging from 1 = *“not at all”* to 7 = “*totally*.” We considered it appropriate to include the coronavirus concern among the other threats given its high salience at the time when this study was conducted. We averaged ratings into a “perceived ecological threat” composite score (Cronbach’s *α* = 0.73). A parallel analysis was conducted (see Supplementary Material); although this analysis supported up to two factors, the inspection of principal component analysis revealed that all items have loading above 0.47 on the first non-rotated factor. Further, the internal reliability was not improved by removing any item.

##### Moral Foundations and Desired Tightness

Participants completed the same measures of binding foundations (Cronbach’s *α* = 0.86), individualizing foundations (Cronbach’s *α* = 0.72), and desired cultural tightness (Cronbach’s *α* = 0.75) as in Studies 1 and 2. Participants also indicated their gender, age, and political orientation as in Studies 1 and 2.

### Analyses and Results

A summary of the means, standard deviations, and correlations between variables is presented in Table 5. As can be seen, perceived threat, the binding moral foundations, and desired tightness were positively and significantly intercorrelated. Perceived threat correlated positively with both the binding [*r* = 0.270, *p* < 0.001, 95% CI (0.158, 0.374)] and individualizing [*r* = 0.353, *p* < 0.001, 95% CI (0.246, 0.450)] foundations. Consistent with previous studies (Jackson et al., 2019), perceived threat also correlated positively with desired tightness [*r* = 0.195, *p* = 0.001, 95% CI (0.080, 0.304)]. The binding foundations correlated positively with desired tightness [*r* = 0.324, *p* < 0.001, 95% CI (0.215, 0.424)]. As in Study 2, the individualizing foundations did not significantly correlate [*r* = 0.101, *p* = 0.088, 95% CI (−0.015, 0.214)], with desired tightness, and as in Studies 1 and 2, the binding foundations were significantly more correlated with desired tightness (Hotelling’s test: *Z* = 2.988, *p* < 0.01) than with the individualizing foundations. Political orientation positively correlated with the binding foundations [*r* = 0.486, *p* < 0.001, 95% CI (0.391, 0.570)], and desired tightness [*r* = 0.268, *p* < 0.001, 95% CI (0.156, 0.372)], and negatively correlated with the individualizing foundations [*r* = −0.232, *p* < 0.001, 95% CI (−0.339, −0.119)].

**Table 5:** Summary of means, standard deviations, and correlations between variables, Study 3.

	M(SD)	1	2	3	4	5	6	7
1. Gender	–	–						
2. Age	22.55(3.06)	−0.026	–					
3. Political orientation	2.75(1.13)	−0.089	0.076	–				
3. Ecological threat	4.89(0.89)	0.289^***^	0.072	0.032	(0.73)			
4. Binding	3.02(0.71)	−0.076	0.059	0.486^***^	0.270^***^	(0.86)		
5. Individualizing	4.77(0.56)	0.226^***^	−0.012	−0.232^***^	0.353^***^	0.158^**^	(0.72)	
6. Desired Tightness	5.64(0.94)	0.057	0.110	0.268^***^	0.195^***^	0.324^***^	0.101	(0.75)

Binding, Binding foundations; Individualizing, Individualizing foundations. Gender was coded as 0 = Male, 1 = Female. ^**^p ≤ 0.01, and ^***^p ≤ 0.001; In parentheses (Cronbach’s alpha). N = 285.

**Figure 2:**
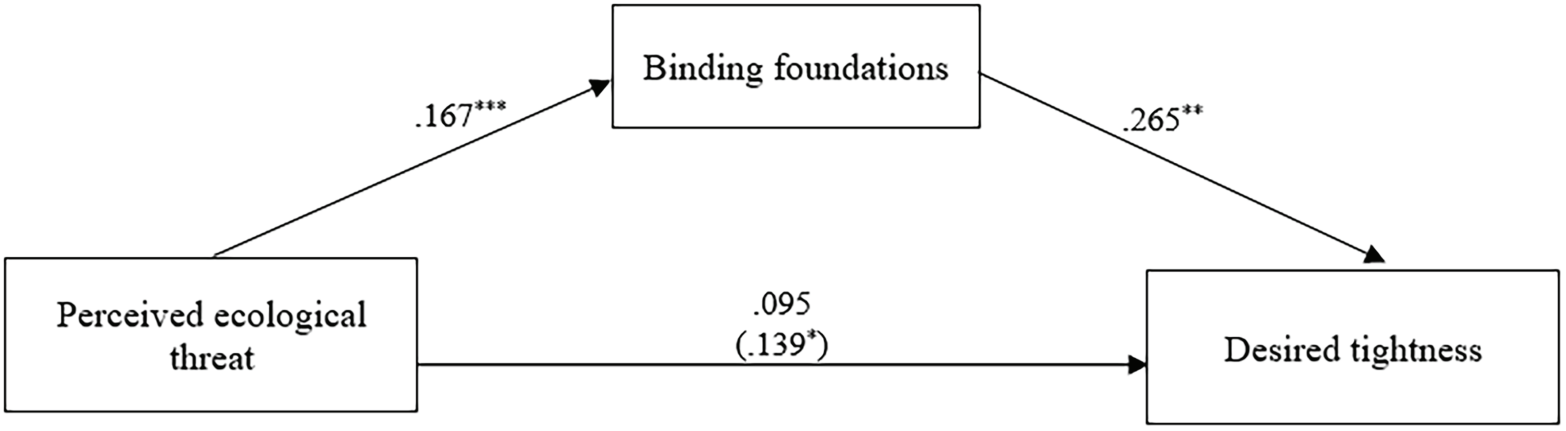
Mediation model. Unstandardized coefficients representing effects of perceived ecological threat on the binding moral foundations and desired tightness. The total effect is in parentheses. To simplify the presentation, the control variables have been omitted. ^*^p < 0.05, ^**^p < 0.01, ^***^p < 0.001.

To test our mediation hypothesis that moral concern for the preservation of the collective as catalyzed by perceived threat may stimulate the desire for stricter norms, we conducted a mediation analysis with PROCESS macro (Hayes, 2018) model 4 with 5,000 bootstrap samples. In order to assess the effects of the binding and individualizing foundations, we included both sets of foundations as mediators. We included gender, age, and political orientation as control variables. In addition, each set of foundations was entered as a covariate when the other set was entered as a mediator; we took this step given the small but significant correlation between the foundations. As can be seen in Table 6 and Figure 2, perceived threat significantly and positively predicted the binding moral foundations, which, in turn, significantly and positively predicted a stronger desired tightness. On the other hand, although perceived threat predicted the individualizing foundations, there was not a significant effect of the individualizing foundations on desired tightness (Table 6). Moreover, and more importantly, the indirect effect of perceived ecological threat on desired tightness through the binding foundations was significant [Indirect effect = 0.044, BootSE = 0.02, 95% BootCI (0.006, 0.095)]; the indirect effect was not significant through the individualizing foundations [Indirect effect = 0.018, BootSE = 0.01, 95% BootCI (−0.013, 0.067)].

**Table 6:** Binding foundations and desired tightness regressed on perceived ecological threat, Study 3.

	Binding			Individualizing			Desired tightness		
	b (SE)	*p*	95% CI	b (SE)	*p*	95% CI	b (SE)	*p*	95% CI
Gender	−0.207 (0.073)	0.005	−0.35, −0.06	0.162 (0.062)	0.010	0.04, 0.28	0.093 (0.113)	0.411	−0.13, 0.31
Age	0.001 (0.011)	0.943	−0.02, 0.02	−0.003 (0.010)	0.770	−0.02, 0.02	0.025 (0.017)	0.153	−0.01, 0.06
Political orientation	0.326 (0.031)	<0.001	0.26, 0.39	−0.179 (0.030)	<0.001	−0.24, −0.12	0.152 (0.056)	0.008	0.04, 0.26
Individualizing	0.299 (0.067)	<0.001	0.17, 0.43	–	–	–	0.117 (0.107)	0.274	−0.09, 0.33
Ecological threat	0.167 (0.042)	<0.001	0.08, 0.25	0.156 (0.036)	<0.001	0.08, 0.23	0.095 (0.066)	0.154	−0.04, 0.23
Binding	–	–	–	0.221 (0.050)	<0.001	0.12, 0.32	0.265 (0.092)	0.004	0.08, 0.45
	*R*^2^ = 0.36, ^Adjusted^R^2^ = 0.35			*R*^2^ = 0.25, ^Adjusted^R^2^ = 0.23			*R*^2^ = 0.15, ^Adjusted^*R*^2^ = 0.13		

*Individualizing = Individualizing foundations; Binding = Binding foundations*. *Gender (0 = Male; 1 = Female), N = 285. Regression coefficients are unstandardized*.

### Alternative Models

Since the SARS-CoV-2/COVID-19 pandemic was a highly salient threat at the time when this study was conducted, we tested the model considering the coronavirus concern as the only threat. We examined both sets of foundations as mediators, and gender, age, and political orientation as control variables. Each set of foundations was also entered as a covariate when the other set was entered as a mediator. The results show that perceived threat of the coronavirus positively and significantly predicted the binding moral foundations (*b* = 0.07, *SE* = 0.03, *p* = 0.013, 95% CI: 0.015, 0.121) which, in turn, positively and significantly predicted desired tightness (*b* = 0.27, *SE* = 0.09, *p* = 0.003, 95% CI: 0.093, 0.448). On the other hand, although perceived threat of the coronavirus also predicted the individualizing foundations (*b* = 0.09, *SE* = 0.02, *p* < 0.001, 95% CI: 0.046, 0.135), there was not a significant effect of the individualizing foundations on desired tightness (*b* = 0.11, *SE* = 0.11, *p* = 0.302, 95% CI: −0.099, 0.318). The indirect effect of perceived coronavirus threat on desired tightness through the binding foundations was significant [Indirect effect = 0.018, BootSE = 0.01, 95% BootCI (0.003, 0.045)]; the indirect effect was not significant through the individualizing foundations [Indirect effect = 0.010, BootSE = 0.01, 95% BootCI (−0.010, 0.035)]. This result indicated that the threat of the coronavirus increases desired tightness *via* binding moral concerns.

We also tested an alternative mediation model in which binding foundations predicted desired tightness *via* perceived threat, with gender, age, political orientation, and the individualizing foundations as covariates, finding a non-significant indirect effect of the binding foundations on desired tightness through perceived threat [Indirect effect = 0.030, BootSE = 0.02, 95% BootCI (−0.012, 0.078)].

### Discussion

In Study 3 we tested our mediation hypothesis that binding foundations may lead to increased desired tightness in the face of a perceived socio-ecological threat. We additionally tested the model considering only the coronavirus threat. Both binding and individualizing foundations were examined as mediators and each set of foundations was entered as a covariate when the other set was entered as a mediator. Control variables were also included in the models. Results showed that perceived threat was associated with an increased desire for tightness *via* the endorsement of binding moral foundations. We additionally tested an alternative model in which the binding moral foundations predicted desired tightness *via* perceived threat, finding a non-significant indirect effect.

## Additional Within-Paper Analysis

We first examined, for each of our studies, the correlations between desired tightness and each moral foundations subscale separately, showing that, across all studies and measurements, the binding subscales correlated with desired tightness more strongly than with the individualizing foundations (see Table 7). We then conducted a meta-analysis across all three of our studies and across the three measurements for both the aggregate (i.e., binding and individualizing foundations) and subscales separately. Given the non-independence of the correlation coefficients between desired tightness at Time 1 and Time 2 (Study 2), we conducted two alternative sets of meta-analyses using the correlation coefficients of desired tightness at Time 1 or at Time 2 separately. We used the META program developed by Kenny (2003) and designed to compute an effect size for each study and pool these effect sizes (i.e., average effect size). As a basic measure of effect size in the meta-analysis, we used the correlation coefficients. Results were weighted by sample size.

**Table 7:** Correlations between Moral Foundations and Desired Tightness and results of Meta-analyses, Studies 1, 2, 3.

					Set 1 including DT at time 1		Set 2 including DT at time 2	
	DT (*Study 1*, *N* = 332)	DT at time 1 (*Study 2*, *N* = 111)	DT at time 2 (*Study 2*, *N* = 111)	DT (*Study 3*, *N* = 285)	Average effect size (Total N = 728)	*t* test of effect size (*df* = 2)	Average effect size (Total N = 728)	*t* test of effect size (*df* = 2)
Binding	0.274^***^	0.504^***^	0.537^***^	0.324^***^	0.36 (*SD* = 0.02)	25.51^**^	0.37 (*SD* = 0.04)	16.49^**^
Authority	0.228^***^	0.475^***^	0.509^***^	0.387^***^	0.36 (*SD* = 0.09)	7.17^*^	0.37 (*SD* = 0.09)	7.30^*^
Loyalty	0.205^***^	0.377^***^	0.409^***^	0.173^**^	0.24 (*SD* = 0.04)	10.04^*^	0.25 (*SD* = 0.05)	8.03^*^
Purity	0.267^***^	0.465^***^	0.485^***^	0.277^***^	0.33 (*SD* = 0.02)	34.11^**^	0.34 (*SD* = 0.03)	21.85^**^
Individualizing	0.128^*^	0.171	0.100	0.101	0.13 (*SD* = 0.02)	10.00^*^	0.11 (*SD* = 0.04)	4.58
Fairness	0.150^**^	0.040	−0.072	0.038	0.08 (*SD* = 0.08)	1.71	0.09 (*SD* = 0.08)	2.02
Care	0.084	0.213^*^	0.188^*^	0.125^*^	0.13 (SD = 0.03)	8.73^*^	0.12 (*SD* = 0.02)	10.51^**^

Binding, Binding foundations; Individualizing = Individualizing foundations, DT = Desired Tightness. ^*^p ≤ 0.05, ^**^p ≤ 0.01, and ^***^p ≤ 0.001.

Additionally, to further estimate the average effect sizes of the association between moral foundations and desired tightness, we performed a within-paper analysis, where participants’ scores were merged into a single dataset (*N* = 728). For homogeneity with Studies 1 and 3, for Study 2 we chose to consider the desired tightness measured at time 1. We first calculated correlations between the two higher-order variables of binding and individualizing foundations and each of five moral foundations (see Table 8). We then performed regression analyses using both approaches of the two higher-order variables of binding and individualizing foundations and of the five sub-dimensions of moral foundations. We used the control variables used in all studies (i.e., gender, age, and political orientation) also including the study number, recoded into two dummy variables [Study 1,3 vs. 2 (1 = 0; 3 = 0; 2 = 1), Study 1,2 vs. 3 (1 = 0; 2 = 0; 3 = 1)], as a fixed effect in the analysis.^5^ Semipartial correlation coefficients are reported to provide an effect size that accounts for the variables in the model. The results of these additional analyses are described below.

**Table 8:** Correlations between the five foundations, pooled data.

	1	2	3	4	5	6	7
1. Binding	–						
2. Authority	0.891^***^	–					
3. Loyalty	0.878^***^	0.667^***^	–				
4. Purity	0.890^***^	0.704^***^	0.663^***^	–			
5. Individualizing	0.272^***^	0.136^***^	0.332^***^	0.253^***^	–		
6. Fairness	0.104^**^	0.013	0.171^***^	0.091^*^	0.833^***^	–	
7. Care	0.340^***^	0.199^***^	0.382^***^	0.321^***^	0.906^***^	0.520^***^	–

^*^p ≤ 0.05, ^**^p ≤ 0.01, ^***^p ≤ 0.001; N = 728.

### Results

#### Meta-Analysis

Results of the meta-analysis (Table 7) showed: for the first set, a more robust average effect size of the binding [average effect size = 0.36 (*SD* = 0.02), *t*(2) = 25.51, *p = 0*.003] compared to the individualizing [average effect size = 0.13 (*SD* = 0.02), *t*(2) = 10.00, *p* = 0.015] foundations; for the second set, a more robust average effect size of the binding [average effect size = 0.37 (*SD* = 0.04), *t*(2) = 16.49, *p* = 0.007] compared to the individualizing [average effect size = 0.11 (*SD* = 0.04), *t*(2) = 4.58, *p* = 0.056] foundations. The results for each moral foundation subscale are presented in Table 7 for both sets and have generally confirmed this trend. Notably, each of the three binding foundations consistently and significantly predicted desired tightness across studies. Results also showed evidence of significant relationships between desired tightness and each of the two individualizing foundations, albeit weak and unstable across studies.

#### Pooled Data

Results of the regression analyses in pooled data across studies (see Table 9), with desired tightness as dependent variable and both the broad categories of binding and individualizing foundations as predictors (along with control variables) showed a positive and significant association of both moral foundations with desired tightness, with a more robust effect of binding [*b* = 0.318, *p* < 0.001, 95% CI (0.209, 0.427)]^6^ compared to the individualizing [*b* = 0.165, *p* = 0.017, 95% CI (0.029, 0.300)] foundations. Afterward, we regressed desired tightness on all the five moral foundations (see Davies et al., 2014; Nilsson and Erlandsson, 2015; Yalçındağ et al., 2019, for a similar approach), along with control variables. Results were reported on Table 10. As can be seen, only the effects of the Authority, Purity and, Fairness foundations on desired tightness were significant and positive. This result will be addressed in discussions.

**Table 9:** Predictive effects of the broad moral foundations and covariates on desired tightness, all studies.

Predictors	*b*	*t*	*p*	95% CI	*sr*
Gender	0.172	2.249	0.025	0.02, 0.32	0.075
Age	0.006	0.649	0.516	−0.01, 0.02	0.022
Political orientation	0.180	5.731	<0.001	0.12, 0.24	0.191
Binding	0.318	5.734	<0.001	0.21, 0.43	0.192
Individualizing	0.165	2.391	0.017	0.03, 0.30	0.080
Study 1,3 vs. 2	0.166	1.582	0.114	−0.04, 0.37	0.053
Study 1,2 vs. 3	−0.032	−0.382	0.703	−0.20, 0.13	−0.013
	*R*^2^ = 0.197, ^Adjusted^*R*^2^ = 0.189				

*Binding, Binding foundations; Individualizing, Individualizing foundations. Gender was coded as 0 = Male, 1 = Female. N = 728. Regression coefficients are unstandardized*.

**Table 10:** Predictive effects of the five moral foundations and covariates on desired tightness, all studies.

Predictors	*b*	*t*	*p*	95% CI	*sr*
Gender	0.177	2.288	0.022	0.02, 0.33	0.076
Age	0.009	0.932	0.352	−0.01, 0.03	0.031
Political orientation	0.178	5.654	<0.001	0.12, 0.24	0.187
Authority	0.249	4.158	<0.001	0.13, 0.37	0.138
Loyalty	−0.041	−0.693	0.488	−0.16, 0.08	−0.023
Purity	0.115	1.960	0.050	0.00, 0.23	0.065
Fairness	0.241	3.250	0.001	0.10, 0.39	0.108
Care	0.004	0.057	0.955	−0.12, 0.12	0.002
Study 1,3 vs. 2	0.165	1.573	0.116	−0.04, 0.37	0.052
Study 1,2 vs. 3	−0.080	−0.923	0.357	−0.25, 0.09	−0.031
	*R*^2^ = 0.212, ^Adjusted^*R*^2^ = 0.201				

*Gender was coded as 0 = Male, 1 = Female. N = 728. Regression coefficients are unstandardized*.

## General Discussion

The main goal of the present research was to provide conceptual and empirical integration of moral foundations theory and cultural tightness–looseness theory. As shown by previous moral foundations theory research (e.g., Koleva et al., 2012), moral foundations permeate individuals’ behavior and their positioning on ideological and social issues. While the individualizing foundations encourage reform of traditional institutions and values to reflect greater equality for individuals and social groups (Graham et al., 2013a), the binding foundations encourage the preservation of traditional values and institutions. Research on cultural tightness–looseness theory shows that the strength of a society’s social norms and the severity of punishment for deviating from those norms is directly related to the degree of threat the society has faced in the past (Gelfand et al., 2011), as tight norms enable people to effectively coordinate to preserve their society in the face of threat. Additionally, perceptions of collective threat to one’s group have been shown to increase individuals’ desire for tighter social norms (Caluori et al., 2017; Gelfand et al., 2017; Jackson et al., 2019). We extended these two bodies of research to examine the effects of moral foundations on desired norm strength using cross-sectional (Studies 1 and 3) and two-wave (Study 2) designs. Consistent with our hypotheses, in all studies, we found that binding moral foundations were positively related to people’s desire for cultural tightness, which also functions to uphold societal rules and norms. In Study 3, we found that the perceived threat was associated with an increased desire for strict norms *via* the endorsement of binding moral foundations. This indirect effect was also found for the coronavirus threat.

Whereas our findings on bindings foundations were generally consistent with our hypotheses, our results for individualizing foundations were somewhat peculiar. The results of our meta-analysis across all studies showed a significant effect for both of our pooled (i.e., both binding and individualizing) foundations in set 1 (i.e., with desired tightness measured at Time 1) but not set 2 (i.e., with desired tightness measured at Time 2). In both cases, there was a more robust average effect size of the aggregated binding foundations compared to the aggregated individualizing foundations, and the trend was generally confirmed for each sub-dimension. A noteworthy finding is that, by combining the data from all the studies, both moral foundations predicted higher scores in desired tightness. Yet, in an analysis without covariates (except for study, dummy-coded), only the binding foundations positively predicted desired tightness. Given these conflicting results, it is possible that there is small relationship between desired tightness and the individualizing foundations that falls outside our hypotheses.

We have a more peculiar result when we observe the five sub-dimensions of moral foundations. In both conducted sets of meta-analyses, we found a significant effect of the “care” foundation on desired tightness, while in the multiple regression analyses performed on the pooled dataset, we found a significant effect of the “fairness” foundation on desired tightness. Both results may have interesting explanations. For example, the care foundation is generally related to compassion, underlying virtues, such as kindness, gentleness, and nurturance (Graham et al., 2013a) and positively correlated with desired tightness in two out of three studies. Potentially, with increased attention to harm caused to others (i.e., care), attention and support for rules that ensure the survival of society can increase. On the other hand, the positive and significant effect of fairness on desired tightness (i.e., in pooled data) suggests that moral attention toward fairness can be linked to a higher desire for tightness, possibly because norms and rules to be respected are important tools to ensure fair treatment and equality. Future research should attempt to replicate and deepen these particular findings, eventually building upon such possibilities.

We must also mention that, in analyzing the five moral foundations, we found a significant and positive effect only of the Authority, Purity, and Fairness foundations on desired tightness. We invite others to take these results with caution, in consideration of the high correlations between the five moral foundations that may have impacted the regression results. We recommend further investigation by future research.

However, it must be recognized that this research is as a first attempt to integrate cultural tightness–looseness with moral foundations theory. Our findings were consistent with the notion that traditional morality (i.e., the binding foundations) motivates a desire for a tight cultural system that maintains the traditions and institutions of a society. This desire may also have a “dark” side, as the binding foundations were previously examined as moral sources of behaviors which are labeled as socially unacceptable, such as racism, blind obedience, and stigma (see Graham et al., 2009). Nevertheless, what we find is also that strong moral concern for the preservation of a society appears to be a reason for invoking compliance with tighter regulations when they are needed to defend it, as in the face of threats, as shown in Study 3.

This research is not without limitations. Any conclusions regarding the individualizing foundations should be taken with some caution also given evidence that these foundations have low internal reliability, as was previously found for both binding and individualizing foundations (e.g., Harper and Rhodes, 2021). As such, the poor reliability of the scales may introduce bias in the relationships between variables.^7^

Additionally, Studies 1 and 3 were conducted with a cross-sectional design, which was addressed with a two-wave design in Study 2. Although linear regression cannot establish a causal relationship on an outcome variable, the results of Study 2 suggest that binding foundations can predict the desire for strong norms at a later time. However, our data do not yield any conclusive causal evidence, and the causal arrow may run in the opposite direction. It is possible that endorsement of cultural tightness causes specific moral beliefs. This deserves further investigation, through experimental studies. Although moral intuitions may be particularly hard to manipulate in a laboratory, future research should attempt to manipulate the level of emphasis people place on moral bases and evaluate their impact on desired tightness. This could inform the causal direction of the relations that we observed. Future research should also manipulate perceptions of ecological threats (see Jackson et al., 2019). Additionally, we point out that our results may vary in other countries, because both moral bases and tightness are realistically influenced by the prevailing national culture. Indeed, some cultures may prioritize respect for the rights and equality of individuals (individualizing focus) while others may prioritize the strengthening of groups and institutions, binding individuals into duties, roles, and mutual obligations (binding focus; Haidt and Joseph, 2007; Haidt, 2008; Graham et al., 2009). Accordingly, conservative norms and standards are present to a greater or lesser extent in cultures (see also Baldner et al., 2020). We therefore might expect the binding concerns to be prevalent in tight systems and elicit support for that cultural system (see Gelfand, 2018; see also Atari et al., 2020, for an investigation of country-level sex differences in moral judgments in relation to cultural looseness). An intriguing direction for research may therefore be to examine in tight vs. loose cultures the patterns found in this work. To conclude, the connection between psychological moral foundations and people’s opinions about cultural systems is an interesting matter of study. This research attempts to advance our knowledge of why and how moral foundations contribute to individuals’ desire for tighter norms in their society.

## Data Availability Statement

The raw data supporting the conclusions of this article will be made available by the authors, without undue reservation.

## Ethics Statement

The studies involving human participants were reviewed and approved by the ethics committee of the Department of Social and Developmental Psychological, Sapienza University of Rome. The patients/participants provided their written informed consent to participate in this study.

## Author Contributions

AP, DDS, and CB executed the studies and performed the statistical analysis. DDS wrote and all authors contributed to the first draft of the manuscript. All authors contributed to the writing, editing, conception, and design of the studies of the final manuscript.

## Funding

This work was supported by Research Grant No. RG118163F3F6F0FE (“Progetto di Ateneo”) awarded by Sapienza University of Rome to AP.

## Conflict of Interest

The authors declare that the research was conducted in the absence of any commercial or financial relationships that could be construed as a potential conflict of interest.

## Publisher’s Note

All claims expressed in this article are solely those of the authors and do not necessarily represent those of their affiliated organizations, or those of the publisher, the editors and the reviewers. Any product that may be evaluated in this article, or claim that may be made by its manufacturer, is not guaranteed or endorsed by the publisher.

## Footnotes

^1^The Moral Foundations Questionnaire by Graham, Haidt, and Nosek and coding are publicly available (including the Italian translation by Bobbio, Nencini, and Sarrica) at www.MoralFoundations.org. Last updated August 26, 2013.

^2^We conducted the regression analyses, for this study as in the following ones, without the control variables. All the analyses without the control variables and results are presented in the Supplementary Material.

^3^Since the dropout rate between the two waves of this study was of 5.1%, we should report information on the possible attrition or mortality-rate effects on our dependent measure. Following Goodman and Blum (1996), we assessed the possible presence of nonrandom sampling bias using multiple logistic regression analysis. This analysis is recommended because it models the probability of being included in one of two response categories (e.g., remaining in or leaving a sample) and considers the relationship among variables. We used a dichotomous variable that distinguished participants who responded in the second wave from those who dropped out as the dependent variable, and all variables of research interest measured in the first wave (i.e., binding and individualizing moral foundations, desired tightness, gender, age, and political orientation) as independent variables. The results of this analysis show no evidence of nonrandom sampling (binding, *b* = 0.48, *p* = 0.636; individualizing, *b* = 0.30, *p* = 0.790; desired tightness, *b* = −0.032, *p* = 0.951; gender, *b* = −2.19, *p* = 0.058; age, *b* = 0.06, *p* = 0.352; political orientation, *b* = 0.20, *p* = 0.657).

^4^Missing values were replaced in SPSS with the series mean. The results remained substantially unchanged without replacing the missing values.

^5^Analyses were repeated without covariates except for study (dummy-coded); results are summarized in the Supplementary Material.

^6^Given that the effect of the binding foundations in Study 1 was not sufficiently large, given our sample size, we re-assessed our effects in the total sample (*n* = 728). According to a sensitivity analysis, and given a sample size of 728 and α and power set to 0.05 and 0.80, respectively, and with seven predictors, we had the power to detect an effect of *f*^2^ = 0.019. Our results showed that the binding foundations had a semipartial correlation of 0.192 with tightness; this is equivalent to an *f*^2^ of 0.046. Therefore, the effect of the binding foundations was sufficiently large to be detected in the total sample.

^7^In order to overcome this problem, an attenuation correction was performed. The estimated true correlation coefficients were similar to the correlation coefficients, so the size difference in the correlation coefficients appears confirmed. It can therefore be considered unlikely that the magnitude of the observed reliability differences influenced the results.
