# Supplementary material for: The Moral Foundations of Desired Cultural Tightness
Source: Front Psychol. 2022 Apr 19;13:739579. doi: 10.3389/fpsyg.2022.739579 (PMC9062776; doi:10.3389/fpsyg.2022.739579)
Supplement: Supplementary file 1 [file Data_Sheet_1.docx]

**Items of desired cultural tightness (Jackson et al., 2019)^[[1]](#footnote-1)^**

My country is currently. . .

1=Not Permissive Enough; 9=Too Permissive

People in my country are currently. . .

1=Overly adherent of my country’s customs; 9= Overly ignorant of my country’s customs

People in my country. . .

1=Follow the rules too much; 9=Don’t follow the rules enough

My country currently has. . .

1=Too many rules; 9=Too few rules

Social norms in my country are. . .

1=Too rigid; 9=Too flexible

People in my country who break the law are currently. . .

1=Punished too often; 9=Punished too rarely

Criminal punishment in my country is currently. . .

1=Too harsh; 9=Too lenient

My country’s norms are currently

1=Enforced too strictly; 9=Not enforced strictly enough

People who don’t conform to the norms in my country are. . .

1=Treated too harshly; 9=Treated too kindly

**Study 1**

We examined predictive effects of moral foundations on people’s desired tightness via multiple regression analysis (R^2^ = .08, ^Adjusted^R^2^ = .07) without including any other control variables. Consistent with the results obtained by including the covariates into the regression equation, without including the covariates the binding foundations predicted higher desired tightness (*b* = .38, *t* = .4.663, *p* < .001, 95% CI: .219, .538), unlike the individualizing foundations (*b* = .09, *t* = .936, *p* = .350, 95% CI: -.104, .294).

**Study 2**

We examined predictive effects of moral foundations measured at Time 1 on people’s desired tightness at follow up via multiple regression analysis (R^2^ = .55, ^Adjusted^R^2^ = .54) without including any other control variables, except for desired tightness at Time 1 to ensure that the effects of the predictors on desired tightness measured subsequently were not explained by their contemporaneous associations. Consistent with the results obtained by including the covariates into the regression equation, without including the covariates the binding foundations positively predicted desired tightness at follow up (*b* = .40, *t* = 3.330, *p* = .001, 95% CI: .161, .637), as well as the desired tightness at time 1 (*b* = .64, *t* = 7.890, *p* < .001, 95% CI: .481, .803), unlike the individualizing foundations (*b* = -.15, *t* = -.964, *p* = .337, 95% CI: -.470, .162).

**Study 3**

We tested our mediation hypothesis (perceived threat on desired tightness trough binding foundations) without including any other control variables. Perceived threat significantly and positively predicted the binding moral foundations (*b* = .21, *SE* = .04, *p* < .001, 95% CI: .124, .302), which, in turn, significantly and positively predicted desired tightness (*b* = .39, *SE* = .08, *p* < .001, 95% CI: .238, .542). The indirect effect of perceived ecological threat on desired tightness through the binding foundations was significant (Indirect effect = .083, BootSE = .03, 95% BootCI [.036, .139]). By including the individualizing foundations as the only covariate in the model, perceived threat (*b* = .193, *SE* = .05, *p* < .001, 95% CI: .098, .288), unlike the individualizing foundations (*b* = .09, *SE* = .08, *p* = .239, 95% CI: -.061, .242), significantly and positively predicted the binding moral foundations, which, in turn, significantly and positively predicted desired tightness (*b* = .39, *SE* = .08, *p* < .001, 95% CI: .236, .541); the indirect effect was still significant (Indirect effect = .075, BootSE = .03, 95% BootCI [.031, .132]).

**Additional within-paper analysis**

The regression analysis of both the binding and individualizing moral foundations on desired tightness was repeated without covariates except for study (dummy-coded). Semi-partial correlation coefficients are presented to provide an effect size that accounts for the variables in the model. As can be seen in Table 11, only the binding foundations predicted significantly (and positively) the desired tightness. Additionally, the regression analysis of both the five moral foundations on desired tightness was repeated without covariates except for study (dummy-coded). The results of this analysis confirmed the results obtained including the control variables in the main analyses: there was a significant and positive effect on the desired tightness of Authority, Purity, and Fairness foundations only (Table 12).

Table 11. Predictive effects of the broad moral foundations on desired tightness, all studies

| Predictors | *b* | | *t* | *p* | | 95% CI | *sr* | |
| --- | --- | --- | --- | --- | --- | --- | --- | --- |
| Binding | .446 | | 8.759 | <.001 | | .35, .55 | .300 | |
| Individualizing | .089 | | 1.366 | .172 | | -.04, .22 | .047 | |
| Study 1,3 vs. 2 | .138 | | 1.296 | .195 | | -.07, .35 | .044 | |
| Study 1,2 vs. 3 | -.121 | | -1.424 | .155 | | -.29, .05 | -.049 | |
|  | | R^2^ = .154, ^Adjusted^R^2^=.149 | | |  |  | |  |

*Note*: Binding=Binding foundations; Individualizing=Individualizing foundations. *N* = 728. Regression coefficients are unstandardized.

Table 12. Predictive effects of the five moral foundations on desired tightness, all studies

| Predictors | *b* | | *t* | *p* | | 95% CI | *sr* | |
| --- | --- | --- | --- | --- | --- | --- | --- | --- |
| Authority | .305 | | 5.066 | <.001 | | .19, .42 | .172 | |
| Loyalty | -.043 | | -.705 | .481 | | -.16, .08 | -.024 | |
| Purity | .171 | | 2.901 | .004 | | .05, .29 | .098 | |
| Fairness | .159 | | 2.139 | .033 | | .01, .30 | .073 | |
| Care | .011 | | .178 | .859 | | -.11, .13 | .006 | |
| Study 1,3 vs. 2 | .131 | | 1.238 | .216 | | -.08, .34 | .042 | |
| Study 1,2 vs. 3 | -.183 | | -2.121 | .034 | | -.35, -.01 | -.072 | |
|  | | R^2^ = .171, ^Adjusted^R^2^=.163 | | |  |  | |  |

*Note*: *N* = 728. Regression coefficients are unstandardized.

| Supplementary Table. Parallel Analysis for Ecological Threat measure (Study 3) |
| --- |

| Factors | Study Eigenvalues | Simulated Eigenvalues |
| --- | --- | --- |
| 1 | 2.73 | 1.22 |
| 2 | 1.21 | 1.13 |
| 3 | .83 | 1.05 |
| 4 | .76 | .99 |
| 5 | .63 | .92 |
| 6 | .45 | .87 |
| 7 | .36 | .78 |

Note: Parallel Analysis was conducted with the principal components

1. In the present research, items have been translated into Italian [↑](#footnote-ref-1)
